# Challenges to the implementation of in situ simulation at HEMS bases: a qualitative study of facilitators’ expectations and strategies

**DOI:** 10.1186/s41077-021-00193-x

**Published:** 2021-11-24

**Authors:** Per P. Bredmose, Doris Østergaard, Stephen Sollid

**Affiliations:** 1 Department of Research, Norwegian Air Ambulance Foundation, Oslo, Norway; 2 Division of Prehospital Services, Air Ambulance Department, Oslo University Hospital, Postboks 414, Sentrum, 0103 Oslo, Norway; 3 Faculty of Health Sciences, University of Stavanger, Stavanger, Norway; 4 Copenhagen Academy for Medical Education and Simulation, Capital Region of Denmark and University of Copenhagen, Copenhagen, Denmark

**Keywords:** Simulation, Prehospital, Air ambulance, Training, Education, In situ, Implementation

## Abstract

**Introduction:**

Facilitators play an essential role in simulation-based training on helicopter emergency medical services (HEMS) bases. There is scant literature about the barriers to the implementation of simulation training in HEMS. The purpose of this explorative interview study was to identify factors that the local facilitators anticipated would challenge the smooth implementation of the program, and their strategies to overcome these before the national implementation of in situ simulation-based training locally, and subsequently, one year after the programme was initiated, to identify the actual challenges they had indeed experienced, and their solutions to overcome these.

**Methods:**

A qualitative study with semi-structured group interviews of facilitators was undertaken before and after one year of simulation-based training on all HEMS bases and one Search and Rescue base. Systematic text condensation was used to extract facilitators’ expectations and experiences.

**Results:**

Facilitators identified 17 themes in the pre-study-year interviews. Pedagogical, motivational and logistical issues were amongst the dominant themes. Other key themes included management support, dedicated time for the facilitators and ongoing development of the facilitator. In the post-study-year interviews, the same themes were identified. Despite anxiety about the perceptions of, and enthusiasm for, simulation training amongst the HEMS crews, our facilitators describe increasing levels of motivation over the study period.

**Conclusion:**

Facilitators prognosticated the anticipated challenges to the successful implementation of simulation-based training on HEMS bases and suggested solutions for overcoming these challenges. After one year of simulation-based training, the facilitators reflected on the key factors for successful implementation.

**Supplementary Information:**

The online version contains supplementary material available at 10.1186/s41077-021-00193-x.

## Introduction

Simulation is well recognised as a useful training method for teams within critical care and emergency medicine [1–4]. This includes prehospital care, where crew-based simulation has been implemented [5–7]. Recommendations for the implementation of simulation-based training have been published, and criteria for success have been suggested [8–10]. Some of these criteria might also apply for prehospital simulation, although the prehospital working situation might differ from in-hospital work with more “down time” waiting for missions. Simulation in prehospital care and helicopter emergency medical systems (HEMS) are often initiated and led by a single enthusiast, which makes such training programmes fragile [5]. Knowledge is sparse about how to implement simulation training in HEMS, and even less about the barriers to such implementation. Facilitators play an essential role in simulation on HEMS bases [11]. However, little is known about their expectations of the role. The facilitator role has been described as a demanding, complex task with a high cognitive load [12]. Participants on a train-the-trainer course for simulation facilitators in the emergency department expressed the view that debriefing is the most challenging part [13]. However, little is known about facilitators’ expectations of the logistics of implementing simulation training on HEMS bases or the pedagogic aspects of facilitating such training.

The purpose of this explorative interview study was firstly to identify what local facilitators anticipated would be the challenges to the implementation of an in situ simulation programme on their HEMS bases and their strategies to overcome these. An in situ simulation-based training was implemented at each HEMS base nationwide [14]. After this programme had been running for one year, the study explored the same facilitators’ reflections on the real challenges to implementation of the program, and how these could be overcome.

## Methods

We used a three-stage explorative design to identify barriers to implementation of in situ simulation training, of the on-call team working in Norwegian HEMS bases. This approach was chosen for practical reasons.

Stage 1 was a session for all participants to identify key topics. Stages 2 and 3 were interviews conducted pre and after one year of simulation training, respectively. The participants were the simulation facilitators. A group-based interview method was chosen to allow group dynamics and participant interaction to elicit key themes [15, 16].

### Participants

Participants in the study were physicians engaged as facilitators in a project to implement in situ on-call simulation at all HEMS bases and one search and rescue (SAR) base in Norway. Both HEMS and SAR are part of the national air ambulance system in Norway and are similar in medical staffing (doctor and assistant) and equipment setup. But whereas HEMS is operated by a civilian operator and mostly runs a three-crew concept where each crew member supports the other, SAR is operated by the Norwegian Royal Air Force with a six-crew concept (two pilots, navigator, technician, HEMS crew member and HEMS physician) where the medical part of the crew is less supported by the rest of the crew [6, 14]. Each facilitator would lead the implementation of the simulation programme on their local HEMS or SAR base.

The local clinical leads at all HEMS bases in Norway, and one SAR base, were invited by e-mail to take part in the program, and to recruit one or two physicians in the air ambulance staff to be trained as facilitators and take responsibility for the local implementation of the program. Because of the differences in crew interaction between HEMS and SAR we decided to only include one SAR base in the project to test if this would influence the implementation of the simulation programme [14]. Sixteen HEMS and SAR physicians were recruited representing all 11 HEMS bases and one of the six SAR bases. Facilitators were required to be clinically active senior prehospital consultants at the HEMS or SAR bases where they would facilitate medical simulation training, but previous simulation experience was not mandatory. None of the authors had any influence on the selection of facilitators. The recruited HEMS physicians were trained as facilitators using the EuSim concept [17].

### Data collection

Data collection was conducted at three different stages during the project.

*Stage 1:* At the beginning of the project, facilitators were invited to a project meeting where the upcoming project was presented. At a brainstorming session, the facilitators were individually asked to name three topics that they expected would be challenging and potentially obstructive for the implementation of in situ simulation at their HEMS base and anonymously write each topic down on post-it notes. The post-it notes were collated, and the facilitators collectively discussed how to cluster and group the topics into themes. The facilitators agreed on three themes: *Motivation*, *frequency* and *delivery* of simulation-based training. The purpose of stage 1 was to identify themes and use these to create the interview guides used in stages 2 and 3.

*Stage 2:* Immediately before the facilitator course, the facilitators were randomly split into two groups of eight. Due to the nature of the small community of prehospital care physicians in Norway, some facilitators would know each other and others not. Two of the authors (PB, SS) conducted a semi-structured interview with each group using an interview guide based on the themes generated in stage 1 and developed by two authors (PB(MD), SS(PhD)) (Appendix 1). The interview guide served to reduce the influence of the interviewers’ pre-understanding, and in addition, the two interviewers would remind each other of the importance of being non-judgmental before the start of the interview [18]. Both interviewers had extensive experience with simulation-based training and were clinically active senior consultants in anesthesiology with extensive air ambulance experience as well as having experience with interviews. The interviewers knew some of the participating facilitators from daily work-elated contact.

*Stage 3:* One year after the start of the simulation training program, all facilitators were invited to participate in a follow-up group interview. Seven facilitators attended. The interview was conducted as a semi-structured interview with one group by the same interviewers (PB, SS) as in stage 2.

**Fig. 1:**
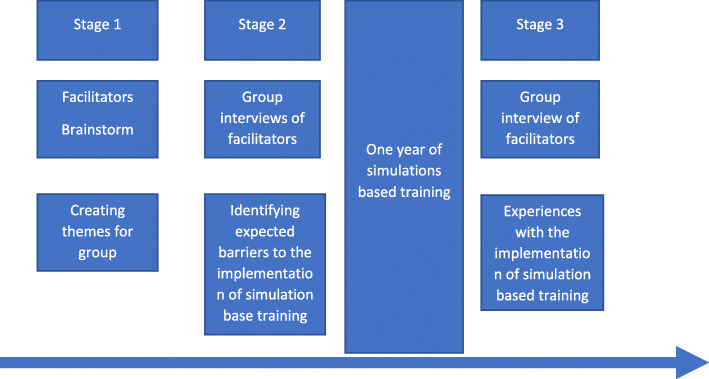
Timeline showing the three different stages of the project

A timeline showing the three different stages of the project is shown in Fig. 1.

### Setting and analysis of interviews

The interviews took place in a closed room during the daytime. The interview guide guided the conversation, but when facilitators raised other issues related to the themes, their spontaneous contribution was encouraged and allowed to be explored further during the interview. The facilitators were also encouraged to follow the thread of previous comments. This is frequently referred to as the “snowball method of sampling” [19]. When a conversation revealed no more new information concerning a topic, the interviewer would prompt them according to the interview guide. The interviews started with an introduction to the research project. All interviews ended with an opportunity for the facilitators to comment and mention anything that they felt had not been addressed.

The interviews were recorded digitally on two independent recording devices. One interviewer (SS) made a coded note of who was talking in the interview. This was subsequently used as an aid in the transcription to identify individual speakers, but each speaker was referenced anonymously in the final transcription and before analysis. The recordings were kept as a safety precaution during analysis but were not used in the analysis process and were deleted upon study completion.

The recorded interviews were transcribed verbatim by a medical student who was not part of the project and received an hourly payment for the job. One author (PB) compared the transcriptions to the recorded interviews to ensure the quality and accuracy of the transcription.

### Data analysis

The data were analysed using Malterud’s “Systematic text condensation” [20, 21]. The data from the interviews at stage 2 were analysed separately from stage 3 data from the post-simulation year.

Two authors (DØ and PB) independently read the transcripts to gain an overview of the interviews and to identify themes. The themes from Step 1 were applied only if appropriate and were not subject to any analysis. The interviews were then annotated to define and identify “meaning units” which covered the themes identified in the previous step. A “meaning unit” is a text fragment/quotation with information about the facilitator’s thoughts. The authors (DØ and PB) discussed and sorted the meaning units into subthemes. Each of these units provided the essence of the subtheme. The meaning units were then synthesised into text. After synthesising, the interviews were reread through to ensure that no information was lost.

We used the COREQ (COnsolidated criteria for REporting Qualitative research) checklist for reporting qualitative research (Appendix 2) [22].

## Results

Sixteen facilitators were included in the interviews before the implementation of the training (stages 1 and 2). The demographic data of the participants are shown in Table 1.

**Table 1:** Demographics of the facilitators

	Median	Range
Age (years)	43	36–52
Experience as physician (years)	14.8	8–23
Experience in prehospital care (years)	7.2	2–17
Experience as simulation facilitator (years)	3.0	0–10

### Interviews before the implementation (Pre-interviews)

Seventeen themes emerged from stage 2 pre-interviews of the facilitators. Nine of these themes were related to the facilitators’ considerations about pedagogical issues in the development, delivery and ongoing improvement of the project. These are shown in Tables 2 and 3.

**Table 2:** Pedagogical considerations expressed in the interviews of the facilitators before the training (*CRM*, crew resource management)

Phases	Themes	Citations
**Development of training**	Consider all team members’ learning needs	Ask all crew members what they think should be included
		Include both medical aspects and technical aspects to involve the pilot and the assistant
		Include CRM aspects, which can contribute to shared situational awareness
		Develop good scenarios
	Development of scenarios	Involve the pilot in the development and assign them precise tasks, e.g. find medication, communicate with the relatives
		Ask the crew members about engaging scenarios
	Motivation of the participants	If the crew see training as useful—they will learn from it
		Make it voluntary and not mandatory to participate
		Involve the most engaged crew members and make them spread the enthusiasm
**Delivery of training**	Level of difficulty	Start with easy scenarios to make crew members familiar with the concept
		Prepare yourself by identifying the individual crew members and think of what they can do
		Keep all crew members motivated by involving them
		Focus on CRM initially and thereafter on medical expertise and skills
		Focus on basic competences in the beginning
	Prepare the participants	Send the theme of the scenario and procedural guidelines in advance
	Psychological safety	Establish a safe learning environment
		What happens in the room stays in the room
		Focus on learning—it is not a test
		Making it safe for the participants will help in making training a part of regular work
	Frequency of training	Take into consideration the shift periods of the individual crew members
		Plan the training at the end of a week, so that the crew know each other
		Consider training once a week except in the busiest periods
		It should be an exemption that training is not conducted
	Faculty training	Training is vital to be able to run the scenarios
		Faculty have high ambitions
**Continuous improvement**	Quality of the training	Crew members will lose interest if we do not secure the quality of the training
		High level of medical expertise to be sure the crew members bring something back
		Be sure that there is not too much repetition

**Table 3:** Individual and organisational factors, barriers and expedient factors expressed in the interviews with faculty before the simulation training period started

	Themes	Barriers	Expedient factors suggested by the facilitators before the simulation period
**Crew members**	Workload	High workload on the base	The facilitator and the crew members have to be flexible
			Accept variation on workload. Plan less training in high seasons and more training in quiet periods
	Expectations and motivation	Pilots are used to frequent simulation of technical skills, and it can be a challenge to involve them in the medical treatment of patients	Involve the pilot in the development of the scenarios
			Clear learning objectives for each crew member
		Pilots are the leader of the crew and can decide that other things should be trained	
		Participants who have a passive role in the training, may lose interest	
		The physician might be the most motivated for training	Focus on both medical, non-technical and technical skills
		The physician is afraid to be tested in their role as a medical expert their knowledge and skills will be exposed to the crew	
		Some pilots will feel exposed. It is expected that they know where things are	You see that the pilot is asked to fetch things, and you will have questions, they have never dared to ask. They ask about the treatment, CRM challenges and other issues that have not been discussed openly before
**Facilitators**	Workload	My own calendar is full	To involve another instructor
			An advantage to have two facilitators, because they can share the workload. A secondary benefit is that they can try both the role of the facilitator and course participant
		The distance to the base is long	Train either before or after being on call myself
		Only one instructor on the base	Facilitation by distance solutions
			Create a facilitator network; a buddy to contact and discuss with would help, could be from another base
	Expectation and motivation	High personal expectations	
		It can be difficult to get started	Be more enthusiastic in the beginning, and then, later, it will be easier for the facilitator
	Expertise in simulation-based training	Lack of routine in/habit of conducting training	Participate in a 3-day instructor course
			It will be easier when you have more routine
			Exchange or visit a facilitator on another base, see how others do it. In addition, you discover the culture at other bases
		Logistical challenge to get the technical things ready	
		Manage to structure the debriefing	Contact other facilitators that can guide you
			Continuous development with the help of other more skilled facilitators from other bases to ensure that I learn from my mistakes. To help me develop my competence
	Development of own competence	Participate in training myself	
		Learning from being a facilitator	The facilitator learns from conducting simulations; they see different solutions and hear reflections. You discover how your colleagues work and you learn a lot from seeing how they solve the tasks
			Learning from colleagues is a benefit—we have to talk more about medical skills in the group on the base. The CRM aspect can also be useful
**Leaders**	Expectation and motivation	Some leaders might be sceptical and do not fully support it	My leaders are very positive—they fully support me and have sent mail stating that simulation is planned and to be seen as equal to other training activities
		Competition with daily missions	In the weekly plan, the facilitator should be free to run the training at least once a week
		Another simulation project is running already	We have to find a way so both projects can run
			If there is maintenance on the helicopter, the crew can still train
	Financial issues	Payment of the facilitators	The project is financed for one year
		At the end of the project, the payment will stop	If the crew members see the training as a positive, a learning experience, they will ask for training after the project has ended

#### Pedagogical issues (Table 2)

Several facilitators mentioned the importance of including the whole crew in the development of training and considering all team members’ learning needs and their preferences for training topics. They felt this was important in order to be able to implement crew resource management (CRM) in the training. It was also suggested that the training should be optional rather than compulsory and use the most positive crew members as advocates for the training.

Some suggested that starting with more straightforward scenarios would ensure a safe start, after which the complexity of the training and the level of medical expertise needed could be increased. It was felt that it was important to establish a safe learning environment, since simulation training might be intimidating for some participants. The creation of a safe learning environment includes focusing on the goal of learning and emphasising that this is not testing. It was mentioned that conducting training at the end of the work week, when all crew members were “settled in” with each other, might make training less anxiety-inducing. The facilitators had high ambitions and mentioned that their own training and education in the simulation was essential to the success of the project.

The facilitators emphasised the importance of maintaining a high level of quality in the scenarios. In addition, they mentioned that the levels of difficulty of the scenarios should be adjusted to the crew members’ level of competence and that repetition of the scenarios should be avoided.

#### Crews

Table 3 shows the remaining eight themes from the interviews of the facilitators, which can be classified as expedient factors, barriers and suggestions for how to overcome these barriers. There were two major themes related to the crew members: workload and expectations/motivation. An excessive workload on the base was considered a barrier to the implementation of simulation training, but an inevitable one which had to be accepted. Some interviewees suggested that it could be overcome by being flexible in scheduling and planning less training in busy periods like mid-summer and holidays. Some facilitators were worried that it might be challenging to involve pilots, who are used to simulation training that focuses on non-technical skills, in this form of training which focuses on medical topics. An expressed fear was that this might worsen if the pilots had a marginal role in the simulations. It was mentioned that any crew members might feel stressed by having their performance exposed and might feel they have not fulfilled others’ expectations of their skills and knowledge.

#### Facilitators and leaders

The workload of the facilitators was also a theme in the interviews. Some facilitators were concerned that they were already busy with full-time clinical work and HEMS shifts. The prospect of having to spend time travelling a long distance to some of the bases was also a concern of some facilitators. One suggested way to overcome this was to involve facilitators from other bases. Another suggestion was to facilitate remotely via video link. The mooted advantages of these solutions were that they would share the workload as facilitators and facilitate mutual support. Some facilitators feared that they would not be able to conduct training, debrief and simulation well enough. These high personal expectations constituted a potential barrier, which they felt could be mitigated by training or collaboration with other facilitators. The facilitators mentioned that they felt that some leaders of the HEMS department might not fully support the project, and other leaders might find that it would compete with already existing simulation training taking place on the base. The costs of the project and the lack of funding for sustainability after the study period were also mentioned.

### Interviews after the implementation (Stage 3)

Four themes emerged: pedagogical issues, timing and planning, crew- and faculty members’ expectations, and motivation (Table 4). The facilitators provided statements representing both barriers and expedient factors. Overall, the facilitators mentioned more expedient factors than barriers.

**Table 4:** The facilitator’s experiences with in situ on-call simulation-based training

Themes	Citations about challenges	Citations about expedient factors
**Pedagogical issues**	Some crew members were sceptical before the simulations	Crew members go smoothly in and out of simulations
		Simulation is not dangerous
	Some crew members have managed to avoid participation in the simulations	The doctors work very independently—good to get feedback
	Some crew members are sceptical to simulation	Big difference between the first simulations and the last
		Positive experience with one team observing another team and providing them feedback afterwards. This was a positive experience—the best moment of learning to receive feedback from a colleague.
		An advantage to receive the scenario before the training
		I expected that it was difficult to get crew members engaged in the simulation and that they would want a high degree of realism. That was not the case.
		In the beginning, the scenarios were easier to make crew members familiar with the setup. To create a safe learning environment. To get the crew into learning mode and not be afraid of showing their weaknesses. Then they were ready to increase their competence
		The more the crew is familiar with simulations, the less they need information beforehand is less
		The more familiar the faculty is with simulations, the easier it is to get the simulation started
		It works, the feedback from the crew is that they have experienced scenarios which they have handled differently after the simulations. The flow and the solutions have not been the same as if we had no training and discussion after the simulations
		Training does not equal simulation—other methods can be used.
		Big-scale scenarios could be useful. Others find it more useful with the small-scale simulations.
**Time and planning**	It is difficult to plan and conduct simulation-based training	It is an advantage if an external facilitator comes and initiates the training
	You spend a considerable amount of time to plan the training, and end up with no simulations on a given day	Best to start at 10-12 and on faculty’s day off.
	There are many interruptions such as visits, inspections and meetings on a busy base	To substitute other types of training with this.
	Crew members mention that they have other on-call duties	Simulation-based training during on-call is not a hindrance to other duties.
**Crew members expectations and motivation**		The pilot and medical assistant have trained to prepare medication and equipment for introducing an arterial line.
		Pilot and medical assistant have used their new skills in critical situations after the training.
		The pilots might have fewer expectations to their own medical skills and hence see it as a less dangerously exposing situation
		They ask for training now.
		The training is well received.
		Crew members like to train, get experiences and reflect
		Medical discussion was needed—“how should this scenario be handled”. An example is provided where the wrong dose of medication and fluids were administered to a child in a simulation.
		The learning gain was considerable—two hours after the training a clinical case where the learning was applied. We knew what we should do.
		The system, organisation and equipment were tested
**Faculty expectations and motivation**	Do we get enough training during the facilitator course?	It was good that we were trained before we started. The tips were useful. Then I had the strength to do it even though the crew was more experienced than me
	It would have been useful to develop the scenarios with another instructor and to be able to discuss the scenario and think of the learning objectives.	It is crucial that you are well prepared—to be able to give the crew something to work with. It is important for the discussions, where to set the level. You must have something with you back as a participant.
	More simulations could be conducted if there were more instructors at the base.	Interesting to see how differently similar scenarios evolve with different crews.
	Can I stay motivated as facilitator?	Faculty has an opportunity to see things from a broader perspective.
		Beneficial to see how others work, see different ways of solving a problem. You get many tips.

#### Pedagogical issues

The facilitators had expected crews would demand a high degree of realism in the scenarios, but this turned out to not be the case. Furthermore, facilitators experienced that as the crews got used to simulations, it was easier to motivate them, less demanding to get started, and less introduction was needed before the simulations. Feedback from crews to the facilitator was mentioned as being an essential tool in the development of the facilitator.

#### Timing and planning for facilitators

The facilitators expressed frustration over spending time planning for simulations and travelling to bases, only for the simulation to be interrupted or not completed. A suggested way to compensate for this was to ensure time is allocated to the facilitator for them to conduct training: participants mentioned the positive impact of having a facilitator that is not on call during the training day, and therefore able to schedule training, and substitute other training forms as needed.

#### Expectations and motivation

The facilitators reported that some crew members were sceptical before simulation and that some even managed to completely avoid participating in simulations during the project period. Some facilitators had experienced profound differences in motivation in the crews from the first to the last simulation in the period and regarded this as a positive development. There was a reported shift in the attitudes of the crew through the project period towards them asking for simulation training, and this was taken as a sign that the training was well-received. The expectations and motivation of the crew members to take part in simulation training increased if recent topics and skills from a simulation scenario were encountered and used in real missions.

Having more than one facilitator at the base was mentioned as a factor that could improve the motivation of the facilitator by relieving the workload and providing a fellow facilitator to spar with as well as increasing the number of simulations offered. It was felt it was important that facilitators were well prepared and able to pitch the scenario and feedback to an appropriate standard of clinical performance. The facilitators found it useful and educational to see how other HEMS crews work. They also mentioned how interesting it was to see how the same scenarios unfold differently when performed by different crews.

## Discussion

In this exploratory study, we found that the pedagogical challenges that facilitators expected were indeed the challenges they encountered. The facilitators described strategies to overcome these challenges. The crews’ positive attitude towards the training was taken as evidence that these challenges were sufficiently mitigated for the scenario training to become a useful educational experience.

The facilitators also expected that a lack of time for conducting simulation training would impede the number of attempted simulations, and this turned out to be true. However, they did not implement all the strategies suggested before the start of the project, such as exchanging ideas between facilitators from different bases. Some strategies were used, and others were not. Although the intention was to give the facilitators individual power to tailor the simulation training to each base, we speculate whether the predetermined structure of this project inhibited this. Despite the availability of project leaders, none of these were consulted during the study period for unknown reasons.

Participating in an initial simulation instructor/facilitator course seems essential, but a focus on ongoing development seems equally important to the participants. This is in concordance with Tariq et al.’s findings, where the complexity of the facilitator role is described [11]. The facilitators emphasised that the success of the simulation-based training depended on expert facilitation, and expressed some anxieties about the new role, and—for some—their lack of experience therein. They suggested that the initial facilitator course should be followed by a continuous development plan for facilitators. Our facilitators did not try to establish a network, although encouraged to do so. However, it was suggested that having more than one facilitator at each base would not only distribute the workload but also contribute to facilitator development. This would be a useful case of micro-network building amongst facilitators: for example, if two facilitators debrief the same scenario (so-called co-debriefing), this interaction could foment mutual development. Co-debriefing has previously been described as a useful tool for facilitator development [23]. However, this was not feasible in the context of this project. Future projects should attempt to pair facilitators with a “buddy” to challenge and stretch their pedagogical skills and role. This would also facilitate scenario development and scenario sharing between bases. Such cooperation could be further enhanced with the implementation of a network between the facilitators.

The facilitators felt it was important that all members of the crew were involved and stayed in their usual professional roles. Our programme was organised in this way a priori. In the interviews before the programme started, the facilitators expressed concerns about how they might engage all members of the crew. The approach that proved successful was starting with simple scenarios and then gradually increasing the complexity of the simulation scenarios. This experience agrees with the findings of Spurr et al. who advocate both the strategy of increasing complexity in the simulations over time and involving the entire multi-professional team members in the simulations [10].

The facilitators reported that the motivation of the crews and their ability to quickly engage in the simulation on the bases increased over time. Motivational factors have previously been described as essential for the implementation of simulation programmes [24]. The facilitators’ lack of experience was concerning, as it could compromise the quality of the training delivered, but the positive attitudes of participants to the training suggest that they felt they largely overcame anxieties mentioned in the pre-project interviews.

The increased motivation and positive attitude towards the simulations may result from the discussion of positive experiences within the relatively small group of staff working at each HEMS base. Sharing positive experiences between bases could further have enhanced this. Facilitators described this sharing of success stories as important for the successful implementation, a finding that is in accordance with one of the eight critical factors listed as essential for successful implementation [8]. The facilitators mentioned the importance of sustainability, which is one of the other factors mentioned by Lazarra [8].

There were concerns amongst the facilitators about the feasibility of continuing the simulation training after the study year. The facilitators mentioned that managerial support for the project would be essential to its viability. The involvement of leadership was similarly mentioned as an essential factor by both Sales and Spurr [9, 10].

### Discussion of the method used

By using an interview-based qualitative method, we captured facilitators’ expectations of barriers to, and expedient factors of, the implementation of simulation-based training. The use of a group-based method might limit the freedom of speech for some participants. However, many of the facilitators knew each other beforehand, and so we think that a safe environment was established in which all participants could contribute. We rationalised that the use of such a homogeneous group with a narrow field of interest was justifiable since the explored topic is narrow too. However, one can speculate whether the homogeneous group excluded the possibilities of gathering different views and thoughts on the topic, which might have emerged if the group were more heterogeneous. During text analysis, there is a chance of information being missed or overlooked. This risk was mitigated by each interview being scrutinised by more than one author. All the authors have experience with simulation training, and these previous experiences can interfere with the conduction of this study. However, one of the authors (DØ) has no prehospital experience. This may have contributed positively to the analysis by introducing a broader perspective since the two other authors are both experienced prehospital care providers, with an existing positive experience with simulation in HEMS systems.

The number of participants in the interviews before the start of the project was higher than in the interview after one year. We did not explore this mismatch but speculate that it might be a result of facilitators’ fatigue during the study period or the general time pressure and workload mentioned by the facilitators. This potential selection bias of participants in the second round may have contributed to a more positive tone in the interviews since the least successful and less motivated facilitators would be less likely to participate.

## Conclusion

The facilitators expected challenges to the implementation of simulation-based training on HEMS bases and suggested strategies for overcoming these challenges before the start of the program. In the one-year follow-up interviews, it was revealed that many of these strategies had not been utilised and that critical barriers to implementation had been experienced, identified, and overcome. The most prominent factors contributing to success were management support, dedicated time for the facilitators to prepare and lead the training, and the need for continuous development within the role as facilitator. Despite fears about the perception of and enthusiasm for the training amongst the HEMS crews, the facilitators described increasing levels of motivation amongst the crews during the study period.

## Supplementary Information

**Additional file 1: Appendix 1**

**Additional file 2: Appendix 2**

## Data Availability

The dataset used during the study is available in an anonymous form from the corresponding author on a reasonable request. The recorded interviews have been deleted.

## Abbreviations

COREQ: COnsolidated criteria for REporting Qualitative research
CRM: Crew resource management
HEMS: Helicopter emergency medical services
SAR: Search and rescue
