# Supplementary material for: Challenges to the implementation of in situ simulation at HEMS bases: a qualitative study of facilitators’ expectations and strategies
Source: Adv Simul (Lond). 2021 Nov 24;6:42. doi: 10.1186/s41077-021-00193-x (PMC8611870; doi:10.1186/s41077-021-00193-x)
Supplement: Supplementary file 1 — Additional file 1: Appendix 1 [file 41077_2021_193_MOESM1_ESM.doc]

# Tema/Struktur for interview

Fasilitator

Interview’er

**Intro/forberedelse**

Skriftlig protokol samt info skal være sendt på forhånd

La kandidaten **lese** innholdet: **Du må bare spørre hvis noe er uklart**.

# Presiser

- rett til å trekke deg når som helst
- anonymitet, (får respondentnummer)
- rett til å få intervjuet slettet (kan ikke endre deler av intervjuet)

**Intervjuet**

# Formalia

Hva er **din formelle og uformelle erfaring/kendskab/uddannelse rundt medicinsk simulation**?

**Hvor lenge har du arbeidet** med dette?

**Hvor** har du drevet med medicinsk simulation?

Hvor arbeider du **nå:** (for båndet)

Hvor **gammel** er du:

Hvor **mange år** har du jobbet som LA lege ?

**Hva skal vi snakke om:**

I denne samtalen vil vi **høre om dine dine tanker knyttet til barrierer ved indføring/opstart af medicinsk simulation i LA tjenesten.**

Vi skal både tale om hvad du mener er barrierer og høre dine forslag til løsning af disse.

Med **medicinsk simulation mener vi indføringen af whole crew simulation i LA tjenetsen i Norge for vagtgående crew.**

Vi har tidligere identificeret flg. 3 hoved grupper af udfordringer :

1. Motivation blandt deltagerne
2. Hyppighed af simulationen
3. At få det realt gennemført

**To typer barrierer til implementering af medicinsk simulation:**

1.
Barrierer på din egen base hvor du skal drive og indføre simulationen.

2.

Indføring på et national plan

**1. Lokale barrierer på din base**

1. **MOTIVATION:**

Hvordan tror du motivationen er bland det vagtgående crew vil være ?

Tenker du at der vil være motivations forskelle mellem piloter, redningsmen og leger ? – kan du uddybe dette ?

Drejer dette sig om generelle holdninger eller holdninger hos enkelt personer i hver faggruppe ?

Kan du give eksempler på ovenstående ?

# FREKVENS:

Hvad tenker du om hyppigheden af simulationer, når det tenkes foregå ugentligt?

Tror du den enkelte lege/redningsman/pilot finder dette for hyppigt?

Er frekvensen en for stor belastning på crew’et?

Er hyppigheden for stor belastning på den/de som skal simulere?

1. **GENNEMFØRING:**

Tror du det bliver en udfordring af få dette gennemført hyppigt nok?
Tror du det bliver vanskeligt at få dette gennemført ud over hele året som projektet skal foregå?

Hvad tror du bliver det vanskeligste i forbindelse med at gennemføre dette?

# Kan du give eksempler på ovenstående ?

#

Kan du komme med tanker og ideer til løsning af de tidligere nevnte udfordringer, når det gælder:
a. Motivation

b. Frekvens

c. Gennemføring

Kan du nevne andre tiltag som kan gøres for at øke implementeringsgraden af medicinsk simulation i LA tjenesten ?

Har jeg **forstått deg rett** når du sier at....

...de største udfordringer bliver:

.... at du tenker flg er en måde at løse det på:

# B. Generelt om indføring af simulation på nationalt plan

Udover de lokale forhold vi allerede har talt om, tror du så der er nogen særlige forhold som kommer til at hindre/reducere indføringen af medicinske simulation på nationalt plan i luftambulansetjenesten i Norge?

Hvad mener du er nødvendigt for at opnå succes med dette projektet ?

tenfor landet?

Avslutning

Synes du **intervjuet gikk greit?**

Er det **noe du føler du ikke fikk sagt** eller som du **vil legge til** før vi nå runder av intervjuet?
